# Effect of different concentrations of pulverized mesocarp of *Citrus paradisi* Macf. on the bromatological characteristics of spray‐dried lemon juice powder

**DOI:** 10.1002/fsn3.679

**Published:** 2018-05-15

**Authors:** Yanilka Alcantara Marte, Yulisa Alcantara Marte, Andrea Escotto Tejada, Gaspar Ros Berruezo

**Affiliations:** ^1^ Department of Food Science and Nutrition Faculty of Veterinary Sciences Regional Campus of International Excellence “Campus Mare Nostrum”, University of Murcia Espinardo Spain; ^2^ Food Technology Department Faculty of Agrifood Science and Environment Universidad ISA Santiago de Los Caballeros Dominican Republic

**Keywords:** bromatology, *Citrus paradisi*, encapsulant, lemon juice, mesocarp, spray‐dry

## Abstract

The aim of this research was to evaluate the effect of different concentrations of pulverized mesocarp of *Citrus paradisi* Macf. as a drying aid on the bromatological characteristics and yield of spray‐dried lemon juice powder. Five concentrations of grapefruit mesocarp encapsulant (0.4, 0.8, 1.2, 1.6, and 2.0% (w / w)) and maltodextrin DE 10 (1.2%, w / w) were evaluated as encapsulant agents. The highest yield (46.76%) was obtained with 1.2% of grapefruit encapsulant. Water activity and ash content were inversely proportional to the added encapsulant concentration. The highest moisture value was obtained with 0.4% and the highest soluble solids with 2.0%. For all treatments, the pH level did not change, except at 0.4% (it was lower). The concentrations of the encapsulants significantly affected the evaluated characteristics, except for the proteins.

## 1 INTRODUCTION

An encapsulating agent, coating material, or drying aid is a compound that forms a thin layer on a surface. They are used to protect a substance or an object from atmospheric humidity and ultraviolet light, among others (Castañeta, Gemio, Yapu, & Nogales, 2011). Currently, there are no coating materials with perfect encapsulation properties for the retention of volatile compounds and the protection against oxidation, or with good emulsifying properties. However, the solubility, hydrophobicity, permeability, and other properties of the material used as a coating agent have a predominant influence on the final product's characteristics (Lozano, 2009).

According to Arellano (2011), a wide variety of coating agents is used in the food industry. The ones used most are polysaccharides (starch, maltodextrin, cyclodextrins, carboxymethylcellulose, gum arabic, and sodium alginate), lipids (waxes and fats), and proteins (gelatin, soy protein, caseinates, buttermilk, and zein) (Gouin, 2004). Beristain (1996) indicates that each of these materials has its limitations; for instance, chemically modified starches have excellent retention of volatile compounds during drying but poor protection against oxidation. On the other hand, partially hydrolyzed starches (maltodextrins, glucose solids syrup, and others) protect against oxidation but lack emulsifying properties. Gum arabic has excellent emulsifying properties and good retention of volatiles; however, it provides limited protection against oxidation of the encapsulated substance, and in recent years, its high cost and scarcity have limited its use.

Fresh citrus mesocarp constitutes around 20%–60% of the whole fruit (Fennema, 1993), depending on its genotype and maturity stage. Citrus mesocarp contains 75%–80% of water, while its main components, calculated in relation to the dry matter, are 44% sugars, 33% cellulose, and 20% peptic substances. It also contains flavonoids, amino acids, and vitamins (Fennema, 1993). However, apart from these nutritional properties, this material is currently wasted during the processing of the grapefruit.

The limited availability of materials used as encapsulating agents and the fact that they have a high cost evidences the need to identify unconventional sources of biomolecules or the like, with functional characteristics similar to those existing. This, along with the nonutilization of by‐products generated in the industrial processing of citrus fruits, led to the development of Alcantara and Escotto's (2014) study, in which an encapsulating agent was obtained from the grapefruit mesocarp.

As a follow‐up to the aforementioned study, the present investigation proposes the evaluation of different concentrations of the encapsulating agent (obtained from the grapefruit mesocarp by Alcantara & Escotto, 2014), which are evaluated in the spray‐drying of Persian lemon juice. This is proposed as an alternative to the existing encapsulants in the market, so as to take advantage of a by‐product generated in the industrial processing of grapefruits, add value, and enable the industrialization and commercialization of a more durable, alternative product that conserves the maximum properties of the encapsulated juice.

Citrus fruits are seasonal; therefore, prices usually fall at the peak of production. For producers, this represents significant losses as the prices do not compensate the production costs. The short shelf life of these species causes losses and can negatively influence trade and consumer confidence (Alcantara & Tejada, 2012). For instance, in a given period, lemons disappear from the market or reach prices so high that most consumers cannot afford. The same happens with other tropical fruits, such as avocado, mango, pineapple, and others. An alternative to mitigate the inconveniences faced by lemon producers and consumers is processing the fruits during the high‐production season. Dehydration of Persian lemon juice was performed due to its nutritional characteristics, short shelf life, and variability in the behavior of prices, given the seasonality of its production.

## 2 MATERIALS AND METHODS

### 2.1 Raw materials

The fruits of *Citrus latifolia* Tanaka were acquired in Santiago de los Caballeros, Dominican Republic, and were used to obtain the drying aid, according to Alcantara and Escotto's (2014) methodology. Maltodextrin DE 10 was used for the encapsulation of the control treatment.

### 2.2 Experimental design

For this study, a completely randomized design was used to evaluate the effect of five concentrations of encapsulant from *Citrus paradisi* (0.4, 0.8, 1.2, 1.6, and 2.0%) as independent variables on yield, physicochemical characteristics (pH level, water activity, and solids soluble), and composition (moisture, protein, ash, carbohydrate content, total phenolic compounds, total flavonoids, and ascorbic acid) of the encapsulated lemon juice. Additionally, 1.2% maltodextrin 10 DE was used as a control. In total, there were six treatments with three replicates, resulting in 18 experimental units.

### 2.3 Process for the lemon (*Citrus latifolia* Tanaka) juice encapsulation

The fruits of *C. latifolia* Tanaka were received in the Food Processing Plant of ISA University and then weighed and selected according to color, size, and appearance (without physical defects). After selection, they were treated with a sodium hypochlorite solution at 100 ppm and allowed to drain for 10 min. Then, they were weighed again and split into two halves. The juice was extracted using manual juicers and filtered using a No. 32 mesh Tyler sieve.

The treatments were prepared by adding 0.5% of tricalcium phosphate as an anti‐adherent (to avoid stickiness and decrease the encapsulated product's hygroscopicity), and the concentration corresponded to the encapsulating agent (i = 0.4, 0.8, 1.2, 1.6, and 2.0% pulverized grapefruit mesocarp; 1.2% maltodextrin DE 10). The percentages were established based on the lemon juice. It was mixed in an Osterizer 4655 electric mixer at full speed for a minute, filtered through a No. 32 Mesh Tyler sieve, to retain any possible particles and avoid obstructions in the atomization needle, and then dried in the Spray Dehydrator YC‐015 SD.

The drying conditions were kept constant: inlet air temperature 130°C, spray air pressure of 3.4 bar, air blower: 4 kg/cm^2^, feed rate: 0.9 L/hr, outlet air temperature 75°C. The particle size was 0.7 mm.

The obtained powder was immediately packed and vacuum sealed in bags. It was stored at 25°C until evaluation.

### 2.4 Evaluated variables

#### 2.4.1 Yield

Yield was evaluated using the methodology described by Lozano (2009), applying the following formula: $\%\text{Yield}=\frac{\text{Grams obtained}}{\text{Grams offered}}\times 100$

The values of grams offered were calculated from the grams of material, and the juice volume was used as the starting material, according to the following equation:

$$\text{Grams offered}=\text{g material}+\text{Vol. Juice (L)}\times \left[\text{Juice}\right]{(}^{\circ }\text{Brix})\times 10$$

#### 2.4.2 Physicochemical variables

Samples were prepared by dissolving 1 g of encapsulated lemon juice in 10 ml of water for the evaluation of the physicochemical variables, except for water activity, moisture, protein, and ash, for which the powder was used directly.

pH: This determination was made by potentiometry at 20°C using a Hach SensION+ 5050T pH meter.

Water activity was determined using the Rotronic HygroPalm (HP‐23), by placing 1 g of encapsulated lemon juice in the cell of the device and waiting for the reading.

Soluble solids were determined using the method 11–15 of Hart and Fisher (1971), using a refractometer (Atago).

#### 2.4.3 Compositional variables

Moisture was determined according to the International AOAC Method (934.01), using the following formula:

$$\text{Moisture Content}\left(\%\right)=\frac{\text{Wet sample weight}-\text{Dry sample weight}}{\text{Wet sample weight}}\times 100$$

Proteins were determined according to Method 2001.11 of the International AOAC.

Ash was determined using Method 923.03 of the International AOAC.

Carbohydrates were determined by applying the phenol‐sulfuric method (Dubois, Gilles, Hamilton, Rebers, & Smith, 1956). The intensity of the orange color was read at 480 nm on a Hach DR 3900 spectrophotometer, against a target prepared in the same manner using water. The amount of carbohydrates present in the sample was calculated from a standard curve prepared with the carbohydrate of interest, treated in the same way as the problem.

Total phenolic compounds were determined using the Folin–Ciocalteu technique (AOCS, 1990). The calibration curve was prepared using a gallic acid standard solution (0.1 mg/ml); to determine the phenols in the sample, the absorbance was measured at 760 nm on the Hach DR 3900 spectrophotometer. The results were expressed as mg of gallic acid equivalent per g sample.

Flavonoids content was determined using Liu et al.'s (2002) method: a calibration curve was prepared using a standard quercetin solution (0.1 mg/ml). The absorbance was measured at 510 nm immediately before 30 min, using the Hach DR 3900 spectrophotometer. The results were expressed as mg of quercetin equivalent per g sample.

Ascorbic Acid: was determined using the method reported by Hung and Yen (2002). The calibration curve was prepared with ascorbic acid, oxalic acid, and distilled water. Absorbance was adjusted to zero, samples were prepared (100 *μ*l aqueous extract, with 900 *μ*l of 2,6 dichlorophenolindofenol), and vitamin C was measured on the Hach DR 3900 spectrophotometer at a wavelength of 515 nm. The results were expressed as mg of ascorbic acid equivalent per gram of sample.

### 2.5 Statistical analysis

The obtained data were evaluated by one‐way ANOVA. Means were separated using Tukey's test (*p *< 0.05). These analyses were performed using Statistix version 8.0. For the representation of the results, the arithmetic mean was used as the central measure ± SD of three replicates; for the means separation, the Tukey test was applied with a 95% reliability.

## 3 RESULTS AND DISCUSSION

### 3.1 Effect of different concentrations of encapsulant retrieved from *Citrus paradisi* Macf. mesocarp on yield of spray‐dried lemon juice

**Figure 1:**
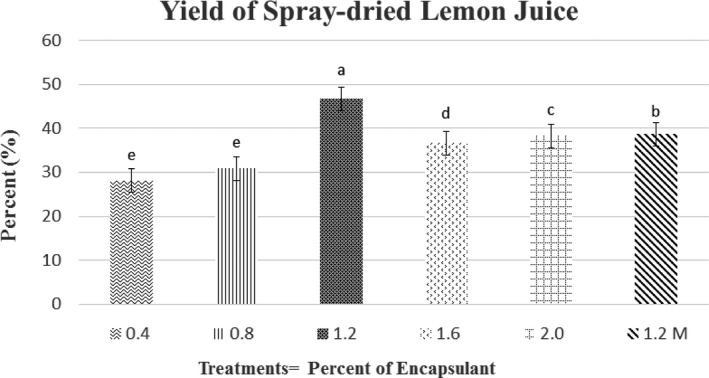
Yield of Spray‐dried Lemon Juice

Figure 1 shows the results of the encapsulated lemon juice yield obtained in this investigation. The yield ranged from 28.15% to 46.76%, corresponding the highest value to the T_12_ treatment, followed by the T_20_ treatment (38.31%) and the control (36.67%). These results can be explained by Caliskan and Dirim's (2013) argument, who state that an increase in the amount of encapsulating agent after a certain interval is not efficient in yields but increases the process cost. Similarly, Fang and Bhandari (2012) found that increasing the maltodextrin concentration above 30% does not have a significant effect on the increase in obtained product yield, considering this concentration as the amount required for a successful drying process of berry juice. In contrast, Mendoza (2015) stated that as the concentration of maltodextrin increases from 20% to 30%, the yield of the product increases because the content of solids in the formulation increases. This is because maltodextrin also causes an increase in the particles size, which makes them less fine; the lowest yield (48.08%) was obtained using 15% of maltodextrin, while the highest value was 74.85% for the treatment, with 30% of this encapsulant.

The yield values found in this study are similar to those obtained by Rivas (2010), who observed a 42% yield for cherimoya juice. However, they are well below the values obtained by Lozano (2009) for microencapsulates of *Opuntia stricta* juice by spray‐drying (51% in the absence of drying aid, and 60–70% with fructo‐oligosaccharides), which is probably due to the high sugar content of citrus juices that causes the product to adhere to the walls of the equipment.

The low values of yield reported in this research can be explained by Lopez, Carvajal, and Millan's (2009) argument, who concluded that a high concentration of total solids in the product is a critical factor in increasing yield in the drying process. The authors estimated that 40% was the minimum soluble solids content expected to get a good yield; therefore, they used 39.95% of maltodextrin (with 21% dextrose equivalents) to dry banana pulp by spray and obtained a 67% yield. Similarly, Sansone et al. (2011) indicate that a pectin concentration lower than 1% in the feed solution is unable to form well‐coated droplets, resulting in the loss of core material during spray‐drying (poor retention of solids after drying).

On the other hand, Romero, Lamuela, Andres, and de La Torre (2001) state that exposure to high temperatures over a prolonged period decreases yield due to the thermal degradation of the compounds. Jun‐xia, Hai‐yan, and Jian (2010) explain that the pH level significantly affects the coacervation between soy isolate protein and gum arabic: pH values of 3.5 or lower result in a significant reduction in coacervate yield.

Moser, Souza, and Nicoletti (2017), using mixtures of whey protein concentrate/maltodextrins and soy isolate protein/maltodextrin, reported that regardless of the protein used, the concentration of the drying aid had a significant effect (*p* < 0.05) on yield (with values between 3.15–75.58%).

### 3.2 Effect of different concentrations of encapsulant retrieved from *Citrus paradisi* mesocarp on the physicochemical characteristics of spray‐dried lemon juice

According to the results of the physicochemical characteristics of encapsulated lemon juice (Table 1), the concentration of pulverized mesocarp from *C. paradisi* Macf. significantly affects the studied variables.

**Table 1:** Results for the physicochemical characteristics of spray‐dried lemon juice

Encapsulant concentration (%)	Hydrogen potential (pH)	Water activity (Aw)	Soluble solids (ºBrix)
0.4	3.041 ± 0.01^b^	0.3653 ± 0.01^a^	8.60 ± 0.53^c^
0.8	3.073 ± 0.01^a^	0.3637 ± 0.00^a^	10.33 ± 0.58^ab^
1.2	3.079 ± 0.00^a^	0.3687 ± 0.00^a^	9.50 ± 0.79^bc^
1.6	3.072 ± 0.00^a^	0.3330 ± 0.01^b^	8.93 ± 0.15^bc^
2.0	3.063 ± 0.00^ab^	0.3310 ± 0.00^b^	11.33 ± 0.58^a^
1.2 Maltodextrin	3.078 ± 0.01^a^	0.3393 ± 0.01^b^	8.93 ± 0.11^bc^

Equal letters in the same column indicate that there is no significant statistical difference (*p* > 0.05) between the means of the evaluated treatments.

Values placed after the ± symbol indicate standard deviation.

Tonon, Brabet, and Hubinger (2008) argue that the final characteristics of a powdered product obtained by spray‐drying depend on some process variables, such as liquid characteristics (solids and viscosity). Zapata, Rojano, and Cortes (2015) ensure that through the drying processes, to which the fruit juices are subjected, various physicochemical changes are generated; they indicate that because the heat directly interferes with spray‐drying, thermal degradation is the most important deteriorating phenomenon.

The results of the present study are corroborated by what was observed by Mendoza (2015), who indicated that all his response variables presented significant differences with respect to the percentage of maltodextrin used to spray‐dry a whey‐based and mango pulp‐based products.

The mean pH values of the treatments of this research (Table 1) are statistically equal to those of the control, with the exception of the T_04_ treatment (juice with 0.4% of encapsulating agent of grapefruit mesocarp); they are also similar to those found by Badillo (2011) for Persian lemon dehydrated in microwaves and dehydrated in trays, corresponding to 3.2–3.5, respectively. Badillo (2011) expresses that acidity increases in dehydrated products because their salts dissociate. The results obtained could also be due to the occurring temperature gradients that cause water diffusion and change its properties in the interior of the foods (Rocca, 2010).

Regarding water activity (Table 1), the results show that the treatments with 1.6% and 2.0% encapsulating agent are statistically equal to the control; whereas, the other treatments are equal to each other, but different from the control. This difference was found by increasing the encapsulant concentration, perhaps because diffusing water through it is difficult. According to Torres (2009), when the additive amount (encapsulant) is low, very unstable particles are formed, which undergo some collapse during the drying process, giving high values of water activity; the lowest values corresponded to the treatments with higher encapsulant concentrations perhaps because in these cases, the particles showed more stability against the temperature, resulting in a more efficient drying.

Mendoza (2015) suggests that at a high spray rate (26,000 rpm) and constant temperature, the increase in the maltodextrin concentration leads to the formation of larger particles, with greater area of heat and mass transfer, decreasing product moisture and its aw. This argument is also confirmed by Torres (2009), who states that drying and concentration processes are used to reduce the water content of a food. Thus, he found that the composition and inlet air temperature influence the value of aw of the dehydrates by increasing solutes concentration and decreasing water activity; he concluded that with a high additive amount (22% maltodextrin) and high inlet air temperature (150°C), lower values (0.165) and therefore more stable solids are obtained.

Additionally, Rodriguez, González, Grajales, and Ruiz (2005) tested for the atomized fig juice, whose dry powder particles were very hygroscopic when the particles had little amount of additive, so that once the powder was formed, being suspended in the humid air, they could be partially hydrated.

The evaluated samples presented aw values between 0.3310 and 0.3687, similar to those obtained by Mendoza (2015), who obtained values between 0.205 and 0.368; these values are also close to those reported by Queck, Chok, and Swedlund (2007) in the drying of watermelon juice (aw ~ 0.3), which according to the mentioned author, allows to consider these food products as microbiologically stable, having a lower water content available for the development of biochemical reactions (aw < 0.6). Marques, Ferreira, and Freire (2007) and Caliskan and Dirim (2013) report that values from 0.2 to 0.4 ensure the stability of the product against reactions of darkening and hydrolytic reactions, lipid oxidation, autoxidation, and enzymatic activity.

On the other hand, Sahin, Dinçer, Torun, Topuz, and Özdemir (2013) reported that the increase in the concentration of encapsulating agents slightly reduced the water activity value, but not significantly. Congruent results were published by Carrillo et al. (2011), Fang and Bhandari (2012), Fazaeli, Emam, Kalbasi, and Omid (2012), and Bustos, Yáñez, and Barragán (2013).

The soluble solids’ results obtained in this research (Table 1) show that the treatments evaluated are statistically equal to control, except for the treatment with 2.0% of encapsulating agent. These values are within the range reported by Kimball (2002) and Mendoza (2003) for dehydrated lemon (8 and 15°Brix). However, these are lower than the value published by Rivas (2010), which corresponds to 24 °Brix; this difference could be because the author used 50% of maltodextrin as an encapsulating agent. On the other hand, Lopez et al. (2009) obtained 46.44 °Brix using 39.95% maltodextrin to dry banana pulp by spray‐drying.

### 3.3 Effect of different concentrations of encapsulant retrieved from *Citrus paradisi* mesocarp on spray‐dried lemon juice composition

Mendoza (2015) asserts that the proportion of nutrient content in the products evidences the influence of the materials and processing process.

Table 2 contains the mean values of the nutritional properties evaluated in encapsulated Persian lemon juices. For the moisture content, it was determined that only the treatment with 0.8% of encapsulating agent is statistically different from the control; the other treatments presented equal or lower moisture percentages, this being a favorable characteristic for the encapsulated product. The highest value was obtained with 0.4% of the encapsulating agent and the control, whereas the lowest value was reported for the concentration of 0.8%. This value is contrary to what was published by Naddaf, Avalo, and Oliveros (2012) who found that the encapsulant that provided the highest moisture protection was 5% maltodextrin, when natural orange juice was spray‐dried using maltodextrin and gum arabic as carrier agents.

**Table 2:** Results of the composition of spray‐dried lemon juice

Encapsulant concentration (%)	0.4	0.8	1.2	1.6	2.0	1.2 Maltodextrin
Moisture (%)	5.67 ± 0.58^a^	3.67 ± 0.58^b^	5.33 ± 0.58^ab^	4.00 ± 0.00^ab^	4.33 ± 0.58^ab^	5.67 ± 1.15^a^
Protein (%)	2.12 ± 0.02^a^	2.09 ± 0.04^a^	2.10 ± 0.06^a^	1.90 ± 0.21^a^	2.01 ± 0.15^a^	1.92 ± 0.06^a^
Ash (%)	16.07 ± 0.23^a^	15.60 ± 0.53^ab^	15.33 ± 0.23^abc^	14.87 ± 0.11^bc^	14.63 ± 0.15^c^	14.73 ± 1.14^bc^
Carbohydrates^a^	15.16 ± 0.62^c^	16.99 ± 0.23^b^	16.70 ± 0.62^bc^	16.95 ± 0.97^b^	19.16 ± 0.20^a^	15.06 ± 0.71^c^
TPC	100.47 ± 0.49^e^	117.22 ± 0.49^d^	122.05 ± 1.65^c^	138.81 ± 0.49^b^	143.00 ± 1.41^a^	91.70 ± 1.15^f^
TF	0.113 ± 0.00^e^	0.178 ± 0.00^d^	0.179 ± 0.00^d^	0.185 ± 0.00^c^	0.307 ± 0.00^a^	0.300 ± 0.00^b^
AA	53.61 ± 0.00^f^	147.74 ± 0.86^d^	180.18 ± 0.00^c^	209.43 ± 2.27^b^	304.07 ± 2.37^a^	113.18 ± 2.87^e^

TPC, Total Phenolic Compounds (mg Gallic Acid Equivalents/g sample); TF, Total Flavonoids (mg Quercetin Equivalents/g sample); AA, Ascorbic acid (mg Ascorbic Acid Equivalents/g sample).

Equal letters in the same line indicate that there is no significant statistical difference (*p* > 0.05) between the means of the evaluated treatments.

Values placed after the ± symbol indicate standard deviation.

a (mg Glucose Equivalents/g sample).

The moisture content of all the treatments evaluated in this study is similar (2–6%) to that reported by Castro (2014) for spray‐dried clarified purple nopal juice and to that observed by Saenz, Tapia, Chávez, and Robert (2009) for drying of nopal juice. They are also congruent with those found by Mendoza (2015), who reported values between 1.48% and 5.84%, the highest corresponding to the lowest concentrations of maltodextrin used; additionally, the product moisture changed from 4.70% to 2.52% by increasing the maltodextrin concentration from 20% to 30%. Similarly, Mishra, Mishra, and Lata (2013) reported that the increase in maltodextrin concentration significantly decreases the moisture content of the powder obtained from amla currant juice (5.6%–3.8%, with maltodextrin values of 5%–9%).

These facts can be explained with what is exposed by Abadio, Domingues, Borges, and Oliveira (2004), who state that in a spray‐drying system, the water content of the feed effects the final moisture content of the powder obtained. The authors explain that the addition of maltodextrin to feed before drying increases the total solids content and reduces the amount of water available for evaporation, which according to Queck et al. (2007), means that powders with lower moisture content could be obtained by increasing the percentage of added maltodextrin.

Table 2 shows that the concentration of the encapsulating agent used does not affect the protein content of the dehydrated product; the treatments evaluated are statistically equal to the control. The values found are higher than reported by Caez and Jaraba (2012) for mango juice encapsulated with maltodextrin (0.59%) and close to that observed by Mendoza (2015) for the powdered product obtained from whey and mango pulp (2.56%).

On the other hand, Naddaf et al. (2012) found that the best protective matrix of proteins was the maltodextrin at 5% and 7% when spray‐drying natural orange juice using maltodextrin and gum arabic.

Concerning the ash content, only the treatment with 0.4% of the encapsulating agent is different from the control and is the one with the highest value (16.07%). In general, the ash content decreased as the concentration of the added encapsulating agent increased.

The ash percentages found are higher than the values obtained by Caez and Jaraba (2012), who reported 0.429% of ash in mango juice microencapsulated with maltodextrin DE 19. These values are also higher than those obtained by Rivas (2010) in enzymatically stabilized cherimoya juice that was microencapsulated using 50% of maltodextrin, corresponding to values between 1.23% and 2.12%. Contrary to what was published by Badillo (2011), who expresses that the ash percentage increases with dehydration because of the desiccation progresses, the water content decreases in Persian lemon dehydration in microwaves and trays, allowing the minerals elements be in higher concentration.

The results obtained for carbohydrates show that only the T_04_ and T_12_ treatments are equal to the control; the others present a greater amount of carbohydrates, indicating that the pulverized grapefruit mesocarp has greater protective effect of this variable.

Table 2 shows that the treatments evaluated are statistically different from each other. The content of total phenolic compounds of the different treatments is greater than the control and as the concentration of encapsulant increases, so does the value of the phenolic compounds; therefore, it could be said that higher the concentrations of the encapsulating agent of pulverized grapefruit mesocarp, the greater protective effect of this variable.

Contrary to the results obtained in this research, Vergara, Guerrero, and Salazar (2009), who evaluated the antioxidant agents of a microencapsulated Jamaica flower extract using mesquite gum at concentrations of 1, 2, 3, 4, and 5%, affirmed that the concentration of mesquite gum did not have a significant effect on the content of phenolic compounds, concluding that all treatments had the same protective effect.

On the other hand, Cardona, Hee, and Talcott (2009) reported losses of 21.5% of Muscadine grape phenolic compounds in a spray‐drying process without the encapsulating agent.

The total flavonoid content for the control was higher (0.300 mg Quercetin Equivalents/g sample) than the other treatments evaluated in the investigation, except for the T_20_ treatment, which had a higher content. These results suggest that increasing the amount of the encapsulant gives higher content of total flavonoids, perhaps because of the protective effect it offers.

Munguía, Castillo, and Elorza (2014) microencapsulated the active compounds of nopal (*Opuntia ficus‐indica*) samples, using seyal gum, Senegal gum, and maltodextrin as encapsulating agents; they determined that the flavonoids of the analyzed samples had greater protection and stabilization/stability when gum arabic and seyal gum are used as wall materials because they provide protection during the encapsulation.

According to the results obtained for ascorbic acid content, Table 2 shows that only the treatment with 0.4% of pulverized grapefruit mesocarp has a lower content of ascorbic acid than the control, so that the other treatments provide better results, which favors the research. The data shown further suggest that the ascorbic acid content also increased as the encapsulant concentration is increased. The treatment that showed greater protection to ascorbic acid was the encapsulating agent of grapefruit with a concentration of 2.0%.

In Gonzalez, Gonzalez, and Rosales's (2011) spray‐dried study of watermelon juice (*Citrullus lanatus* Thunb) using maltodextrin and gum arabic as encapsulating agents at concentrations of 0.5% and a mixture of both at the concentration of 0.5%, they determined that the treatment corresponding to 0.5% of the mixture of maltodextrin DE 10 and gum arabic (1: 1) w / w was better; they stated that in this treatment, the volatile compounds in the spray‐dried powdered watermelon product did not show significant difference against the original extract.

The findings in this study are also confirmed by Liu (2014), who states that the pectin–starch relation influences the physical and functional properties of the encapsulated ascorbic acid microparticles; their results suggested that the proportion of starch–pectin influenced the encapsulation efficiency of ascorbic acid more than the type of starch.

## 4 CONCLUSION

The yield of the encapsulated lemon juice is significantly influenced using different concentrations of pulverized grapefruit mesocarp (0.4, 0.8, 1.2, 1.6, and 2.0%). The concentrations of pulverized mesocarp from creole grapefruit (*C. paradisi* Macf.), used in this research, significantly affect the evaluated physicochemical characteristics (pH, water activity, and soluble solids) of the encapsulated lemon juice. Raising the encapsulant concentration increases the pH level and decreases the water activity of the encapsulated juice.

Using different concentrations of pulverized grapefruit mesocarp (0.4, 0.8, 1.2, 1.6, and 2.0%) has a significant influence on the composition of the encapsulated lemon juice (percentage of moisture, ash, carbohydrate content, total phenolics, flavonoid content, and ascorbic acid content), except for the protein content of the juices. As the concentration of drying aids increases, so does the content of bioactive components. From the doses used in this research, it was determined that 1.2% of grapefruit mesocarp could be used as an encapsulant for lemon juice during spray‐drying.

## ETHICAL STATEMENTS

This study does not involve any human or animal testing.

## CONFLICT OF INTEREST

None declared.
